# Cost analysis in implementation studies of evidence-based practices for mental health and substance use disorders: a systematic review

**DOI:** 10.1186/s13012-021-01094-3

**Published:** 2021-03-12

**Authors:** Diana M. Bowser, Brandy F. Henry, Kathryn E. McCollister

**Affiliations:** 1 Heller School for Social Policy and Management, Brandeis University, 415 South St, Waltham, MA 02453 USA; 2 School of Social Work, Columbia University, 1255 Amsterdam Ave, New York, NY 10027 USA; 3 Department of Public Health Sciences, University of Miami Miller School of Medicine, 1120 NW 14th St., CRB 1019, Miami, FL 33136 USA

**Keywords:** Economics, Costs, Implementation, Evidence-based practices, Behavioral health

## Abstract

**Background:**

This study is a systematic literature review of cost analyses conducted within implementation studies on behavioral health services. Cost analysis of implementing evidence-based practices (EBP) has become important within implementation science and is critical for bridging the research to practice gap to improve access to quality healthcare services. Costing studies in this area are rare but necessary since cost can be a barrier to implementation and sustainment of EBP.

**Methods:**

We followed Preferred Reporting Items for Systematic Reviews and Meta-Analyses (PRISMA) methodology and applied the Consolidated Health Economic Evaluation Reporting Standards (CHEERS) checklist. Key search terms included: (1) economics, (2) implementation, (3) EBP, and (4) behavioral health. Terms were searched within article title and abstracts in: EconLit, SocINDEX, Medline, and PsychINFO. A total of 464 abstracts were screened independently by two authors and reduced to 37 articles using inclusion and exclusion criteria. After a full-text review, 18 articles were included.

**Results:**

Findings were used to classify costs into direct implementation, direct services, and indirect implementation. While all studies included phases of implementation as part of their design, only five studies examined resources across multiple phases of an implementation framework. Most studies reported direct service costs associated with adopting a new practice, usually summarized as total EBP cost, cost per client, cost per clinician, and/or cost per agency. For studies with detailed analysis, there were eleven direct cost categories represented. For five studies that reported costs per child served, direct implementation costs varied from $886 to $9470 per child, while indirect implementation costs ranged from $897 to $3805 per child.

**Conclusions:**

This is the first systematic literature review to examine costs of implementing EBP in behavioral healthcare settings. Since 2000, 18 studies were identified that included a cost analysis. Given a wide variation in the study designs and economic methods, comparison across studies was challenging, which is a major limitation in the field, as it becomes difficult to replicate studies or to estimate future costs to inform policy decisions related to budgeting. We recommend future economic implementation studies to consider standard economic costing methods capturing costs across implementation framework phases to support comparisons and replicability.

Contributions to the literature

- Implementation research of evidence-based behavioral health interventions has grown dramatically in the past 10 years; however, costing methods and types are still not standardized which makes cost comparisons across studies difficult.
- We describe the types of costs and costing methods which have been used to date in implementation research of evidence-based behavioral health interventions.
- Results of this analysis inform future studies in identifying appropriate costs and methods to include in research which will improve rigor and replicability.

## Introduction

### Background

Cost analysis is broadly applied through economic evaluations of treatment interventions and related programs for mental health/substance use disorders (SUD), often as part of an effectiveness/cost-effectiveness study. Recently, cost analysis has become an important area within the implementation science field, which focuses on the translation of research into practice and disseminating methods and applications of evidence-based practices (EBP) on a broader scale [1]. Implementation science has developed to understand the factors that facilitate the adoption of EBP to assist in bridging the research to practice gap, and to improve the accessibility and quality of health services [2]. Health services focused on mental health and SUD, across the lifespan, fall under the broader area of behavioral health. Research and evaluation of the implementation of EBP in behavioral healthcare is a rapidly expanding area of importance, particularly since EBP adoption in this area has lagged behind that in traditional healthcare settings. Economic studies in this area are especially important since costs can be a barrier to implementation of EBPs in behavioral healthcare agencies [3], and funding/payment for behavioral health services are often separate from other health services.

In their seminal article, Proctor et al. specifically called for implementation research to include costs as an outcome [4]. The inclusion of costs in implementation research is important because the costs of implementing EBP can drive decision-making by service providers. Such decision-making impacts whether an EBP will be adopted or sustained, which ultimately impacts service quality and patient outcomes [5]. However, despite the importance of including costs, a systematic review of implementation research on practice guidelines found that only about a quarter of implementation studies include costs [6]. This is relevant because the bulk of costing studies in implementation research focuses on practice guidelines [5].

Implementation studies within the broader health service delivery field that included economic evaluations were summarized in a recent systematic literature review by Roberts et al. [7]. This review identified 30 studies, most of which were hospital-based. More than half of the included studies used cost-effectiveness analyses, while smaller proportions used cost utility analyses or cost-consequence analyses. Measured costs included staff training, development of new care processes/pathways, and patient/caregiver-related costs. While this study provided a useful overview of the economic tools used in implementation research, it did not specify how these tools have been used in the area of nonhospital-based behavioral health services or how costs are collected across implementation phases.

### Study aims

The extent to which researchers have included costs in their implementation studies of EBP in behavioral health services is not known. Additionally, the costing methods applied in these studies have not been systematically described. We address this gap by conducting a systematic review of the literature to understand the types of costs and costing methods which have been used to date in implementation research of evidence-based behavioral health interventions in the USA and Canada. We focus on these two countries due to their similarities in the epidemiology of behavioral health disorders, overlapping related health policies [8], and flow of both patients and programs across the border [9]. We exclude other countries due to the unique nature of the US healthcare financing [10]. Addressing these issues can elucidate areas of potential growth in how implementation researchers incorporate cost analysis as a core measure. While costs are an important driver, they certainly do not overshadow the importance of patient outcomes, which is why we focus only on the implementation of EBP, which have already been linked to quality services.

## Methods

### Identification of studies

We conducted an initial systematic review (February 2019) and updated the search prior to submission (April 2020). We investigated costing studies in behavioral health implementation research using Preferred Reporting Items for Systematic Reviews and Meta-Analyses (PRISMA) methodology [11] and checklist [12]. We developed key search terms through reviewing search terms in MeSH® [13]. Key search terms included four areas: (1) economics, (2) implementation, (3) EBP, and (4) behavioral health. Included articles contained at least one key term from each area. Specific key terms were economic, cost, implementation, evidence-based practice, behavioral health, mental health, mental illness, mental disorder, psychiatric, addiction, drug abuse, substance use, and drug use. Key words related to specific types of substances (e.g., opioids) were not included to reduce complexity.

We based our search strategy on suggestions from psychology and economics published best practices for systematic literature reviews [14, 15]. Terms were searched within article title and abstracts in the following databases: EconLit, SocINDEX, Medline, and PsychINFO. We did not include Embase, as we were not interested in biomedically or pharmaceutically related research, and excluded studies focused only on drugs. Included articles contained at least one key term from each area. Abstracts were downloaded into Excel and independently screened by two of the authors to determine if they met inclusion and exclusion criteria. Full-text screening was conducted for all articles meeting inclusion criteria. Full-text screening was also conducted for articles where insufficient information was provided in the abstract to determine if criteria were met. The Boolean search and selection criteria are listed below. There is no published review protocol.

((TI economic) OR (TI cost) OR (AB economic) OR (AB cost)) AND ((TI implementation) OR (AB implementation)) AND ((TI evidence based practice) OR (AB evidence based practice)) AND ((TI behavioral health) OR (AB behavioral health) OR (TI mental health) OR (AB mental health) OR (TI mental illness) OR (AB mental illness) OR (TI mental disorder) OR (AB mental disorder) OR (TI psychiatric) OR (AB psychiatric) OR (TI addiction) OR (AB addiction) OR (TI drug abuse) OR (AB drug abuse) OR (TI substance use) OR (AB substance use) OR (TI drug use) OR (AB drug use))

### Inclusion criteria

The following are the inclusion criteria:

1. Articles that address implementation studies of behavioral health services which incorporate costing (formal economic analysis was not required for inclusion)
2. Studies published after 2000
3. Studies only if original research is presented (quantitative or qualitative data)
4. Community-based studies (outpatient level of care or services provided to people residing in the community, rather than a hospital)
5. Peer-reviewed articles
6. Published in English
7. Research was conducted in the USA or Canada
8. Studies providing services to people of any age

### Exclusion criteria

The following are excluded in the study:

1. Published before 2000
2. Editorials, newspapers, and other pieces of popular media
3. Dissertations or theses
4. Book chapters, book reviews, proceedings, government and/or organization reports, and other publications from the gray literature
5. Systematic reviews
6. Protocols without preliminary data
7. Case study
8. Non-implementation-based studies
9. Published in a language other than English.
10. Studying a system outside the USA or Canada
11. Studies based in hospitals
12. Did not include costing data
13. Focusing solely on comparing medications

### Data extraction and analysis

**Fig. 1:**
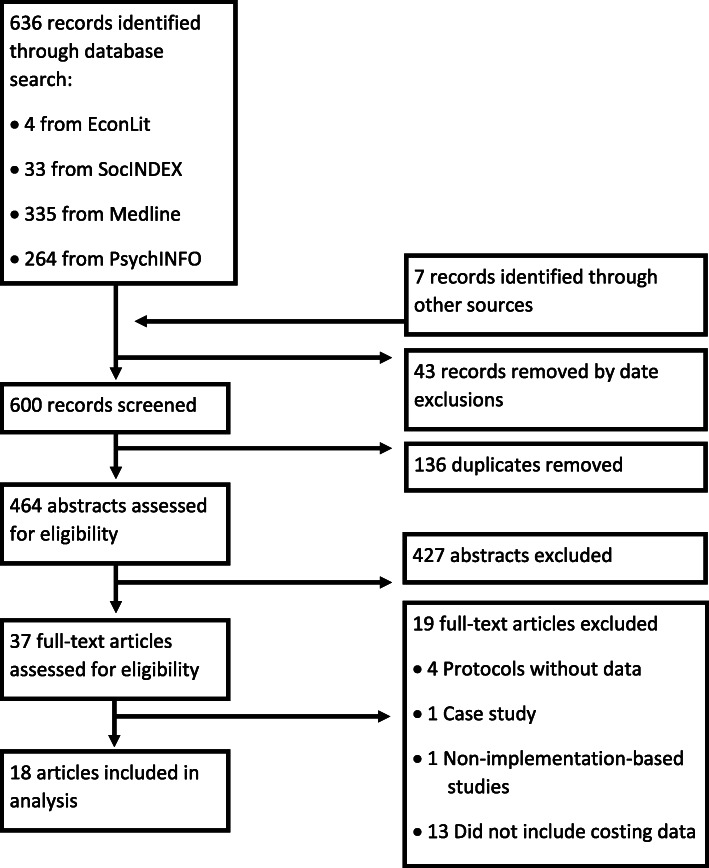
Search results using PRISMA

As shown in Fig. 1, the initial abstract search returned 636 articles (four from EconLit, 33 from SocIndex, 335 from Medline, and 264 from PsychINFO). Seven articles were added based on review of references during full text review. After excluding articles based on date of publication and those that were duplicates, a total of 464 unique abstracts were screened independently by two authors. Authors also screened articles individually for risk of bias, but did not exclude any articles based on this issue. This was reduced to 37 articles after applying inclusion/exclusion criteria to abstracts. The final number of included articles after full-text reviews was 18.

The following characteristics were extracted from all included studies: authors, year published, journal, area of behavioral health, type of EBP, sample size, implementation framework, economic methodology, costing approach, costing perspective(s), cost categories and specific resources, cost analysis results, and economic study design. Thematic analysis was conducted to summarize similarities and differences across studies. Content analysis was used to identify how these characteristics overlapped within included studies, such as identifying costing perspectives and resource categories that overlapped across studies. Content analysis classified three categories of costs for the purpose of the study: direct implementation, direct service, and indirect implementation. For a subset of studies, direct and indirect implementation cost results were extracted and reported. In studies where summary cost estimates were not provided, we used the aggregated cost results (as presented) combined with patient/family/client sample sizes provided within the articles to approximate summary costs (i.e., cost per child served) to support comparisons across studies.

The Consolidated Health Economic Evaluation Reporting Standards (CHEERS) checklist [16] was used to assess the quality of the articles. This checklist was designed to provide a standardized reporting system for economic evaluations of health interventions. Since we included other implementation studies with costing components, in addition to economic evaluations, we reported the overall percent of included items on the checklist (removing not applicable items from the denominator, rather than a score). We eliminated several checklist items that were not relevant for costing studies that did not also include an economic evaluation. We converted CHEERS checklist quality scores used in previous research [17, 18] to a percentage. Previous studies applied the following scoring categories: excellent (24 out of 24 points or 100%), good (at least 18 out of 24 points or 75-99%), average (at least 12 out of 24 points or 50-74%), and poor (11 or fewer out of 24 points or 0-49%).

## Results

Table 1 provides the list of included studies with details on study design, type of EBP, target population, implementation framework, and economic methodology. Only one of the included articles was published before 2010 [19]. Most articles (12 articles) were published in specialty journals focusing on behavioral health. The remainder were published in specialty journals related to public health (four articles [20–23]), children’s services (one article [19]), or implementation science (one article [24]). Several studies focused on specific demographic groups [children with substance use disorders, mental health disorders, or trauma (six articles [19, 23–27]), adolescents/youth with mental health disorders (two articles [21, 28]), justice-involved youth (two articles [29, 30]), geriatric mental health (one article [20]), and veteran’s mental health (one article [31]). Studies focusing on specific diagnoses included: alcohol use (one article [32]), addiction (one article [33]), co-occurring disorders (one article [34]), mental health disorders (two articles [22, 35]), and serious mental illness (one article [36]).

**Table 1:** Included study sample description

Author	Year	Journal	Area of Behavioral Health	EBP	Sample Size (unit of analysis)	Implementation Framework	Phases (pre-implementation, implementation, sustainability)	Cost Type	Economic Study Design	Economic Perspective
Dopp et al	2018	Behavior Therapy	Behavioral health & juvenile justice	Multisystemic therapy	1,869 (children)	Context and Implementation of ComplexInterventions framework	Implementation	Direct Service Costs	Cost-benefit analysis	Taxpayer
Dopp et al.	2017	Psychological Services	Youth mental health	Trauma-focused cognitive behavioral therapy	574 (clinicians) & 1,410 (youth)	Learning Collaborative	Combines pre-implementation & implementation	Direct Implementation Costs and Indirect Implementation Costs	Cost effectiveness analysis	Organization
Fisher et al	2017	Population Health Management	Geriatric Mental Health	Remote learning and mentoring program	154 (clinicians)	None	Implementation	Direct Service Costs	Direct costing	Organization
Greer et al	2014	Administration and Policy in Mental Health	Children's mental health	Trauma-focused cognitive behavioral therapy	90 (children)	None	Implementation	Direct Service Costs	Direct costing	Organization
King et al.	2018	Translational Behavioral Medicine	Alcohol Use	Screening and brief intervention for alcohol misuse	3 (health systems in 3 states)	Sustainment Evaluation using Program Sustainability Assessment Tool	Sustainability	Qualitative Cost Categories	Direct costing	Organization
Knusden & Roman	2015	Journal of Substance Abuse Treatment	Addiction treatment	Various evidenced-based treatments and practices	307 (organizations)	Typology of level of EBP adoption	Pre-implementation, Implementation, & Sustainability	Qualitative Cost Categories	Direct costing	Organization
Lang & Connell	2017	Journal of Behavioral Health Services & Research	Children's Behavioral Health	Trauma-focused cognitive behavioral therapy	10 (clinics)	Implementation Cost Survey	Pre-implementation, & Implementation	Direct Implementation Costs	Direct costing	Organization
Lee, Walker & Bishop	2016	Psychiatric Services	Mental health & juvenile justice	Psychiatric practice guidelines	3 (facilities)	None	Implementation	Direct Service Costs	Direct costing	Facility
McKee et al	2013	Journal of Dual Diagnosis	Co-occurring disorders	3 month integrated treatment program	155 (individuals with co-occurring disorders)	Transformation, implementation and present evaluation	Implementation	Direct Service Costs	Direct costing	Organization
Palinkas et al.	2017	Health Research Policy and Systems	Adolescent Mental health	Various evidenced-based practices	75 (agency chief executive officers/ program directors)	Agency leadership model of implementation	Pre-implementation, Implementation, & Sustainability	Qualitative Cost Categories	Direct costing	Organization, Staff, Consumers
Rollins et al.	2017	Administration and Policy in Mental Health	Veteran's mental health	Assertive community treatment	32 (VA mental health management teams)	Comparison of three fidelity measures: on-site, phone, and expert-scored self-report	Implementation	Direct Implementation Costs	Direct costing	Organization, Staff
Roundfield & Lang	2017	Psychiatric Services	Child Traumatic Services	Trauma-focused cognitive behavioral therapy	14 (community mental health agencies)	Sustainment Phase Costs	Combines pre-implementation & implementation	Direct Implementation Costs and Indirect Implementation Costs	Direct costing	Organization
Saldana	2014	Implementation Science	Child services	Unspecified	75 (sites)	Stages of Implementation Completion	Pre-implementation, Implementation, & Sustainability	Qualitative Cost Categories	Direct costing	Organization
Shi et al	2016	American Journal of Managed Care	Depression	Quality measures	17 (multi-stakeholder community coalitions)	None	Implementation	Qualitative Cost Categories	Direct costing	Society
Stewart et al	2016	Psychiatric Services	Mental health	Unspecified	49 (agency/ policy leaders)	Implementation Science	Pre-implementation, Implementation, & Sustainability	Qualitative Cost Categories	Direct costing	Organization
Suarez et al.	2014	Hawai'i Journal of Medicine & Public Health	Children's mental health	Gender-responsive trauma-informed care	72 (youth)	None	Implementation	Direct Service Costs	Direct costing	Society
Swain et al	2010	Community Mental Health Journal	Serious mental illness	Assertive community treatment, Family psychoeducation, Integrated dual disorders treatment, Illness management and recovery, Supported employment	49 (sites)	None	Sustainability	Qualitative Cost Categories	Direct costing	Organization
Swenson et al	2000	Children's Services: Social Policy, Research, and Practice	Maltreated children in state custody	Interagency collaboration	45 (families)	None	Implementation	Direct Implementation Costs and Direct Service Costs	Direct costing	Interagency Collaboration

Table references: [19–36]

Most articles (15 articles) investigated the implementation of a single EBP. However, some studies (three articles) considered implementation of multiple practices [21, 33, 36]. EBPs delivered to patients included multisystemic therapy, screening and brief intervention for alcohol use, trauma-focused cognitive behavioral therapy, gender-responsive trauma-informed care, assertive community treatment, integrated treatment, family psychoeducation, integrated dual disorders treatment, illness management/recovery, and supported employment. EBPs that were delivered to providers focused on remote learning and mentoring, improved practice guidelines, quality measures, and enhanced interagency collaboration.

Studies varied in sample size and unit of analysis. Four studies measured EBP adoption on individual patient outcomes. The sample size in these studies ranged from 72-1,869 people; however, three of the studies had a sample size between 72-155 [23, 25, 34]. One study measured the impact on families (N=45) [19]. Another study measured outcomes for patients (N=1,410) and clinicians (N=574) [28]. Yet another study measured outcomes for clinicians only (N=154) [20]. Three studies focused on management staff and/or teams, with sample sizes ranging from 32 (teams) to 75 (staff) [21, 31, 35]. Eight studies measured EBP impact on the organization/agency or system and had sample sizes ranging from 3-307 organizations [22, 24, 26, 27, 30, 32, 33, 36].

### Implementation frameworks and costing approaches

Most articles (11 articles [21, 24, 26–29, 31–35],) reported using an implementation framework, although there were no studies that reported using the same framework. This is indicative of the broad set of implementation frameworks that exist within the field, and the complexity of internal and external factors that must be considered in evaluating an implementation process based on type of EBP, setting, and target population. The Consolidated Framework for Implementation Research (CFIR) provides a point of reference as it includes a comprehensive list of constructs (intervention characteristics, agency/staff characteristics, outer and inner contexts within which the agency operates, and the implementation process itself) [37]. In this framework, cost is mentioned as a key measure of the implementation process, but no details are provided as to recommended approaches, measures, or data sources for estimating implementation costs. In this review, about half (five articles) of the included studies were developing a framework, or specific tools, for understanding implementation processes and/or phases. These tools/frameworks focused on costs (two articles [26, 33]), EBP sustainability (two articles [27, 32]), and implementation stages and fidelity (one article [24]). The remaining studies adopted existing implementation frameworks to conceptually guide their research.

Most implementation frameworks follow a phased structure, generally including pre-implementation, implementation, and sustainment activities and corresponding evaluation metrics. Cost analyses must define an analytic perspective, and in implementation research, two key perspectives are the agency/provider/organization that would be deciding to adopt an EBP and the payer, sometimes the agency itself, health insurance plan, or Medicaid/Medicare. In cost-effectiveness research, the Second Panel on Cost-Effectiveness in Health and Medicine recommends adopting the healthcare sector and societal perspectives, both of which are broader than a provider or payer perspective. The societal perspective includes costs to the healthcare system, other systems (e.g., justice, education, social services), and patient/caregivers. The cost analysis perspective defines the relevant resources and monetary conversion factors for estimating costs across implementation phases. General categories of implementation costs include direct labor costs, indirect labor costs, and nonlabor costs [5]. Direct labor costs reflect personnel time spent implementing an EBP, including time spent training and delivering services to clients. Indirect labor costs refer to personnel time outside of clinical services, such as time spent recording case notes or administrative duties. Nonlabor costs essentially cover all other resources required for implementation such as contracted services, equipment, supplies, licensing, building/space costs, and administrative overhead. Studies use other many terms to characterize costs such as direct implementation costs, direct service costs, opportunity costs, accounting costs, as well as fixed costs and variable costs. All implementation phases (pre-implementation, implementation, and sustainment) have some combination of these costs. Pre-implementation costs would typically include labor costs associated with meetings and trainings during preparation and planning phases, as well as one-time expenditures on equipment or supplies to prepare for the implementation phase. Implementation costs would include labor costs for those delivering services or performing administrative roles, and recurring expenditures on supplies, equipment, space, communications. Sustainment costs would include the cost of implementation resources that are required to continuously support the EBP.

For the purposes of this review, we classify three categories of costs guided by the presentation of costs in the included studies: direct implementation costs, direct service costs, and indirect implementation costs (Table 2). As shown in Table 2, direct implementation costs capture the actual expenditures incurred by agencies/providers implementing the EBP for pre-implementation and implementation activities such as meetings, trainings, purchasing training manuals, and travel. Direct implementation costs were calculated mainly through activity-based costing, tallying the time spent on implementation activities and applying reported or imputed salary data to estimate the direct labor costs for implementation activities. Direct service costs are those costs associated with billable healthcare and other services resulting from the implementation process or specific EBP activities. About half of the included studies calculated direct service costs using Medicaid or other claims data [19, 20, 22–26, 29, 30, 34].

**Table 2:** Direct and indirect cost estimations for implementation studies

Cost type	Definition	Examples of measurements
Direct costs of implementation	Direct costs incurred as a result of EBP implementation including training of staff, purchasing of manuals/instruction aids, travel expenses, meeting, calls, data collection and management, site visits, supervision	Hours/minutes on implementation-related activity*(Base salary per hour/min + Fringe/benefit) + Travel (Airfare, hotel, ground transportation)
Direct service costs	Direct costs of billable healthcare and other related services as a result of implementation activity	Claims data analyzed for services related to specific population in EBP
Indirect costs of implementation	Lost time spent on implementation activities rather than usual clinical/professional activities	Time spent on implementation activities* hourly direct cost rate for normal billable activity; Indirect costs are classified as on-going (regular meetings) as well as one-time events (trainings)

Indirect implementation costs capture the opportunity cost for agencies due to lost revenues and/or time spent on implementation activities rather than standard (pre-implementation) clinical activities that could be billed and reimbursed. Indirect costs were estimated using the amount of time that a clinician reported spending on implementation activities, multiplied by the reimbursement rate for their billable activities [27, 28]. Two of the 18 studies measured the indirect costs associated with implementation of EBPs [19, 24]. Seven studies do not report cost figures, but rather used primary data collection and qualitative methods to summarize specific examples of both direct and indirect implementation costs incurred at the agency during the implementation process [21, 22, 24, 32, 33, 35, 36]. Almost all studies (sixteen) applied direct costing, although one study used cost-benefit analysis [29], and another used cost-effectiveness analysis [28]. Different costing perspectives were represented across the 18 studies. Eleven studies described using a provider (i.e., organizational) perspective, with one other study also including costs incurred by organization staff (but not reimbursed directly) [31] and another including costs incurred by staff as well as consumers [21]. One study applied a facility perspective (one location of a multi-location organization) [30], one reported an interagency perspective (e.g., representing costs incurred across collaborating agencies) [19], and one reported a taxpayer perspective [29]. Two studies did not fully describe their perspective but provided enough detail to infer that they used a societal perspective [22, 23].

Using the cost categories defined in Table 2, we noted five studies examining costs within all phases of implementation (including sustainment) based on the adopted framework [19, 21, 24, 33, 35]. Three studies examined only pre-implementation and implementation phases, all of which focused on trauma-focused cognitive behavioral therapy. These studies measured either direct implementation costs, direct services costs, and/or indirect implementation costs [26–28]. Eight studies included costs aligned only with the implementation phase [20, 22, 23, 25, 29–31, 34]. All of these studies measured direct services costs, except one study, which measured direct implementation costs [31]. The remaining two studies were focused on the sustainability phase, and provided qualitative measurement of direct implementation costs [32, 36].

As shown in Table 3, there was a wide variation in the types of direct cost categories in the implementation costing studies. We identified eleven distinct categories of direct implementation costs captured across these five studies. Eight of these categories captured direct costs attributable to staff time spent in implementation activities such as trainings, meetings, on-site consultations, follow-up calls, supervision, project management, and preparation time. While the actual name of the cost categories differed slightly across the studies, the objective of including these eight direct cost categories was similar. For example, on-site meetings are called “learning sessions” [28] in the study of community-based implementation of trauma-focused cognitive behavioral therapy, and “on-site assessor reviews” in another study [31]. The purpose of including this cost category is to understand how much time is devoted to learning how to appropriately implement the EBP. The three final cost categories captured time devoted to data activities required of implementation studies or the actual purchase of data equipment as well as transportation expenses and training materials purchased. No single study included all 11 cost categories. One study of the implementation of a trauma-focused cognitive behavioral therapy program for youth included nine of the eleven cost categories [26]. Another study examining three specific methods for assessing implementation activities (on-site, phone, and self-report), included four cost categories only [31]. One article focused implementation costing on training and travel expenses only [19].

**Table 3:** Direct implementation cost categories

Cost categories	Lang & Connell 2016	Dopp et al. 2017	Roundfield & Lang 2016	Rollins et al. 2017	Swensen et al. 2000
Community readiness and consultation		X			
Training (including initial orientation and senior leadership training)	X	X	X		
Consultation calls during implementation (with clinicians, senior leaders and, social workers)	X	X	X	X	X
On-site meetings (learning sessions, assessor reviews)		X		X	X
Supervision	X		X		X
Implementation team meetings	X				
Administrative/project coordination	X	X	X		X
Non-billable implementation preparation time	X		X	X	X
Data requirements (collection, management and infrastructure)	X	X	X		
Training materials	X	X			X
Transportation expenses	X	X		X	X

Table references: [18, 23, 27, 28, 34]

Studies reporting quantitative cost results across the highlighted costing categories are summarized in Table 4. Since it was difficult to compare cost results between studies given the variation in the type of EBP implemented and the number of cost categories included, the results were summarized by type of cost (direct versus indirect), type of EBP, and average costs (per child, per clinician, per collaborative). The results show that even within similar cost categories and types of costs, there was still considerable variation. For example, among the five studies that reported direct implementation costs [19, 26–28, 31], four reported direct implementation costs per child, which ranged from $886 per child to $9470 per child. For the three studies that implemented the same EBP (trauma-focused cognitive behavioral therapy for children), direct implementation cost per child varied between $886 and $2742 [26–28]. Two studies reported indirect implementation costs ranging from $897 per child to $3805 per child [27, 28].

**Table 4:** Direct and indirect implementation costs

		Average Total Direct Implementation Costs			Average Indirect Intervention costs		
Authors	EBP	Per Child, mean (SD)	Per Clinician/ staff member mean (SD)	Per agency /Learning Collaborative mean (SD)	Per Child, mean (SD)	Per Clinician/ staff member mean (SD)	Per agency /Learning Collaborative mean (SD)
Dopp et al. (2017)^b^	Trauma-focused cognitive behavioral therapy	$886	$2,176	$96,168	$3,805	$9,345	$412,635
Roundfield & Lang (2017)	Trauma-focused cognitive behavioral therapy	$1,000 ($811)	$2,353 ($1,909)	$34,371 ($27,898)	$897 ($889)	2110 ($2,091)	$30,822 ($30,563)
Lang & Connell (2017)	Trauma-focused cognitive behavioral therapy	$2,742 ($1,615)	$11,659 ($2,930)				
Rollins et al. (2017)	Assertive community treatment	$2,579^a^ ($804)					
Swenson et al (2000)	Interagency collaboration	$9,470 ($201)					

Note: ^a^Per assessment, ^b^Standard Deviation not provided

Three studies examined costs across phases of implementation: pre-implementation, implementation, and sustainment. All three of these studies also reported average per-child costs. One study combined pre-implementation and implementation direct costs totaling $886 per child [28]. A second study reported average direct implementation costs per child of $1,000 and indirect implementation costs per child of $897 per year during the sustainment period [27]. In another study, while the authors reported staff costs separately for pre-implementation and implementation, they report an aggregate summary of the average direct implementation costs ($2,742 per child) for both phases together [26].

Results from the CHEERS checklist (Table 5) indicate that, on average, studies included 87% of applicable items from the CHEERS checklist, which corresponds to good quality. The highest quality (excellent) studies include all applicable items [32, 33, 35, 36]. All studies, except one, included at least 75% of applicable items (good quality), with the one exception including only 65% (average quality) [23]. The most common item that was not included was the discount rate, with only 8% of studies including that item. Several other items from the CHEERS checklist related to cost-effectiveness analysis and were largely not applicable. These items included measurement of effectiveness, measurement of valuation, and characterizing uncertainty/heterogeneity.

**Table 5:** CHEERS quality rating

CHEERS item	[29]	[28]	[20]	[25]	[32]	[33]	[26]	[30]	[34]	[21]	[31]	[27]	[24]	[22]	[35]	[23]	[36]	[19]	Mean
1 Title^a^	1	1	N/A	1	N/A	N/A	1	1	N/A	N/A	1	1	N/A	1	1	N/A	N/A	1	100%
2 Abstract	1	1	1	1	1	1	1	1	1	1	1	1	1	1	1	0.5	1	1	97%
3 Background	1	1	1	1	1	1	1	1	1	1	1	1	1	1	1	1	1	1	100%
4 Population	1	1	1	1	1	1	1	1	1	1	1	1	1	1	1	1	1	1	100%
5 Setting	1	1	1	1	1	1	1	1	1	1	1	1	1	1	1	1	1	1	100%
6 Perspective	1	1	1	1	1	1	1	1	1	1	1	1	1	0.5	1	0	1	1	92%
7 Comparators	1	1	1	1	1	1	1	1	1	N/A	1	1	1	1	N/A	1	1	1	100%
8 Time horizon	1	1	1	1	1	1	1	1	1	1	0	1	1	1	N/A	1	1	1	94%
9 Discount rate	1	0	0	0	N/A	N/A	0	0	N/A	N/A	0	0	0	0	N/A	0	N/A	0	8%
10 Choice of health outcomes	N/A	1	0.5	N/A	1	1	N/A	1	1	N/A	1	N/A	N/A	1	N/A	1	1	1	95%
11 Effectiveness	N/A	1	N/A	N/A	N/A	N/A	N/A	N/A	N/A	N/A	N/A	N/A	N/A	N/A	N/A	N/A	N/A	N/A	100%
12 Measurement /valuation	1	0	N/A	N/A	N/A	N/A	N/A	N/A	N/A	N/A	1	N/A	1	1	N/A	N/A	N/A	N/A	80%
13 Resources & costs	1	1	1	1	N/A	1	1	1	N/A	N/A	1	1	N/A	1	N/A	0	N/A	1	92%
14 Currency/ price conversion	1	1	1	1	N/A	N/A	1	0.5	0	N/A	0	1	1	0.5	N/A	1	N/A	0.5	73%
15 Model choice	1	1	0	1	1	1	0	1	1	1	0.5	1	1	1	N/A	0	1	1	79%
16 Assumptions	1	1	0	1	1	1	0	0	0	0	0	1	1	1	N/A	0	1	1	59%
17 Analysis	1	1	0	1	1	1	0	1	1	1	1	1	1	1	1	1	1	1	89%
18 Parameters	1	1	1	1	1	1	1	1	1	1	1	1	0	1	1	0.5	1	1	92%
19 Incremental costs/outcomes	N/A	1	1	1	1	1	1	1	0	1	1	1	0	1	N/A	1	1	1	88%
20 Uncertainty	0	1	N/A	N/A	N/A	N/A	0	N/A	N/A	N/A	N/A	N/A	N/A	N/A	N/A	0	N/A	1	40%
21 Heterogeneity	1	1	N/A	1	N/A	N/A	N/A	1	N/A	N/A	N/A	N/A	N/A	1	N/A	N/A	1	1	100%
22 Discussion	1	1	1	1	1	1	1	1	1	1	1	1	0.5	1	1	1	1	0.5	94%
23 Funding	0	1	1	1	1	1	1	1	0	1	1	1	1	1	1	1	1	0	83%
24 Conflicts	1	0	1	0	1	1	1	1	1	1	1	1	1	1	1	1	1	0	83%
Mean score	90%	88%	76%	90%	100%	100%	75%	88%	76%	93%	79%	95%	81%	91%	100%	65%	100%	82%	87%

^a^Studies with N/A for "Title" were not focused on economic evaluation, but included costing; 0.5 indicates partial fulfillment of the criteria

## Discussion

### Overview of the field

This is the first systematic literature review to examine cost analyses within implementation studies of EBP for behavioral health services. The literature review identified 18 studies published since 2000 that included a formal cost analysis. Most of this work has been published in recent years (16 articles since 2013). Given the increase in cost analyses within implementation research of behavioral health EBPs, this study provides important context for the current state of the field, including a summary of findings, and we offer suggestions for how the field might standardize economic concepts and measures to increase translatability and support data harmonization going forward. In particular, the large number of not applicable categories from the CHEERS checklist may indicate a need for a new checklist of costing as part of implementation research.

### Review of results

We found several studies proposing the development of an implementation research framework, but no studies using a previously published framework. This finding resonates with a recent study by Wensing and Grol (2019) that identified a large number of implementation frameworks within the field, but very little consistency across contextual determinants [38]. Our findings describe and categorize existing methodologies for calculating direct costs of EBP implementation, direct costs of healthcare services, and indirect costs of EBP implementation. These three categories of cost most closely align with how cost results were presented in the included studies, but they diverge slightly from standard cost categories in the economic evaluation literature. For instance, economic analyses of treatment interventions typically include start-up costs (aligned with pre-implementation costs) and intervention management/operational costs (aligned with implementation and sustainment costs). Within these categories, resources are typically characterized as sunk costs (e.g., start-up), variable costs (dependent on number of patients or clients), time-dependent (recurring costs during the year to support implementation), and societal costs (e.g., opportunity costs of subsidized/donated resources, staff and participant’s time and travel costs).

We identified a wide variation in study type and costing methods, thus limiting the comparability across cost analysis results. Further complicating comparability is the possibility that there could be some double counting in the direct implementation and direct service cost categories if staff time is being reported as a provider expense as well as a billable service cost. Only one study included both of these categories [19], and the methods and results were not detailed enough to determine the magnitude of double counting. However, this limitation is important for future comparisons of implementation costs. Despite these challenges, we were able to highlight 11 distinct categories of direct implementation costs that are captured in sufficient detail across five of the studies. This review also highlights the importance of estimating the indirect implementation costs—or opportunity costs—in both seeking and providing services. Opportunity cost in this context refers to the lost revenue from billable clinical services due to implementation activities. Several of the implementation studies calculated opportunity costs. These calculations are extremely important from the provider perspective as they represent the large monetary investment that is made in implementing EBPs and other programs at the expense of direct billing for providing healthcare services.

Regarding the presentation of summary cost estimates, three studies examined the same EBP (trauma-focused cognitive behavioral therapy), which facilitated comparison. However, even among these studies there was wide variation in reported costs, which may suggest that costs are substantially impacted by factors outside of the implementation process. Some of the reported costs were also relatively high (as much as $3,805 per child), and may be inflated due to the inclusion of fixed startup costs (i.e., pre-implementation costs that would not vary with the number of children served) and smaller caseloads. In fact, one of the included studies highlights this in reporting that per child and per session incremental implementation costs are highest in the start-up phase and decrease over time as more children are served [26]. However, as is known, the cost of healthcare in the United States is highest in the world, and this includes the costs of behavioral healthcare. As pharmacotherapy is a large component to behavioral healthcare, relatively high drug prices in the United States could be an important driver. For example, one study reported the annual per capita cost of treating an adult for a behavioral health disorder to be $2,745 [39]. Other research has identified that factors outside implementation can impact costs. For example, behavioral health patient caseloads and state level Medicaid and Child Health Insurance Program (CHIP) funding may be associated with lower implementation costs and better retention in behavioral health services programs for justice-involved youth [40].

### Implications for future research

Given the wide variation in the types of factors included in implementation research it is difficult to make comparisons across studies. This is a major limitation of implementation research focusing on behavioral health services, as it becomes difficult to replicate studies or use studies to estimate future costs to inform policy decisions related to budgeting. With increasing emphasis on the economics of implementation science [38], the field could benefit from adopting standardized guidelines, especially for costing perspectives, approaches (e.g., activity-based costing), instrumentation, and categorization of cost components. In the broader economic evaluation literature, standard methods for cost analysis are established [41, 42]. Recently, standardized approaches for economic analysis in behavioral health were also described [43]. As implementation science grows, similar guidelines need to be developed. Without standardization and harmonization of data across studies, it is difficult to fully assess how the evidence of effectiveness and economic impact generalizes from one study to a broad area of practice or research.

Based on our findings, we recommend that future studies include more details about the specific activities and resources associated with an implementation process, so that other researchers and policy makers can anticipate what costs will be incurred in changing existing or adopting new practices. We also recommend that studies include measures of environmental context such as treatment capacity, available funding (i.e.: block grants) and urbanicity, all available from public sources, to better capture how outside factors may impact the costs of implementing EBPs [44]. As shown in two studies, urbanicity was related to behavioral health EBP delivery [45, 46]. Beyond impacting the effectiveness of the EBP, contextual factors impact costs directly. For example, salaries may be impacted by geography due to higher costs of living in certain areas.

### Limitations and strengths

While this study adds substantially to the literature by describing the state of the field to date, there are still several limitations. For example, since there was very little overlap between studies in the types of EBP being implemented or outcome measures, we were unable to conduct a meta-analysis. Our findings were also focused on implementation studies of evidence-based behavioral health services, and therefore do not extend to other health-related services. Further, only one included study reported both direct implementation costs and direct service costs [19], which limited our ability to estimate the ratio of implementation to service costs. Concerns about double counting staff time or other resources across direct implementation and direct service cost categories could not be explored in this study given the level of detail provided. Additionally, we also focused only on nonhospital-based services within the United States and Canada, therefore excluding hospital-based services. Since healthcare systems and behavioral health epidemiology in countries outside the United States and Canada are quite different, this decision likely improved the specificity of our findings. However, we are unable to generalize outside of this geographical area. Additional research focused on single-payer healthcare systems, like in the United Kingdom, that would illuminate the role of costing in both policy decisions and implementation of services. While these choices likely improve selection process, there are likely other important areas of research conducted outside the scope of this work. Future studies should aim to describe findings in these areas. Had we included hospital-based services and health services more broadly, it is likely that there would have been greater variation in costing approaches and results.

## Conclusion

Implementation research on EBPs in behavioral health has grown significantly in the last several years. However, the field has not yet standardized the use of economic methods or measures. Recommendations based on our findings include moving toward standard cost measures to facilitate cross study comparisons and the potential for results to drive policy decisions.

## Data Availability

Data can be obtained by replicating the key search terms in the listed academic databases.

## Abbreviations

EBP: Evidence-based practices
PRISMA: Preferred Reporting Items for Systematic Reviews and Meta-Analyses
CHEERS: Consolidated Health Economic Evaluation Reporting Standards
SUD: Substance use disorders
CFIR: Consolidated Framework for Implementation Research
CHIP: Child Health Insurance Program
